# The Effect of Cyberbullying, Abuse, and Screen Time on Non-suicidal Self-Injury Among Adolescents During the Pandemic: A Perspective From the Mediating Role of Stress

**DOI:** 10.3389/fpsyt.2021.743329

**Published:** 2021-11-12

**Authors:** Tjhin Wiguna, Kusuma Minayati, Fransiska Kaligis, Raden Irawati Ismail, Erik Wijaya, Belinda Julivia Murtani, Kent Pradana

**Affiliations:** ^1^Department of Psychiatry, Faculty of Medicine, Universitas Indonesia – Dr. Cipto Mangunkusumo General Hospital, Jakarta, Indonesia; ^2^Faculty of Psychology, Universitas Tarumanegara, Jakarta, Indonesia

**Keywords:** COVID-19, adolescents, Indonesia, cyberbullying, abuse, screen time, stress, non-suicidal self-injury

## Abstract

Adolescence is often a period of turmoil. The COVID-19 pandemic has increased adolescents' difficulty due to mental health consequences that may affect their developmental milestones. This study constructed and empirically tested a theoretical model of three predictive factors (cyberbullying, abuse, and screen time) and stress as the mediating factor in adolescent non-suicidal self-injury (NSSI). Structural equation model (SEM) analysis was applied to investigate stress as a mediating factor in the relationship between adolescent NSSI and cyberbullying, abuse, and screen time. This cross-sectional study used a “crowdsourcing” sample collection method to recruit 464 adolescents aged 11–17 years who were administered a questionnaire comprising scales on cyberbullying, abuse, screen time, stress, and NSSI. All scales had construct reliabilities ranging from 0.759 to 0.958. SEM statistical analysis was performed using Lisrel version 8.8 (Scientific Software International, USA) for Windows (Microsoft Corporation, Redmond, WA, USA). The mean (± SD) age of the cohort was 14.61 ± 1.65 years, and consisted of 66.7% females. Secondary high school was the highest educational background (58%). The study found that cyberbullying and abuse were direct positive predictors (critical *t*-value for the path > 1.96; *p* < 0.05) of adolescent NSSI; however, screen time did not have any direct relationship. Furthermore, stress was a significant full mediating factor of screen time and a partial mediating factor of cyberbullying and abuse in the relationship with adolescent NSSI (critical *t*-value of the path = 5.27; *p* < 0.05). Cyberbullying, screen time, and abuse with the mediating effect of stress could explain 48% of the variance in adolescent NSSI (*R*^2^ = 0.48). Adolescent mental health prevention and promotion programs need to be redesigned during the current COVID-19 pandemic to manage their stress and minimize the mental health consequences of cyberbullying, abuse, and inappropriately increased screen time.

## Introduction

The coronavirus disease 2019 (COVID-19) pandemic has continued for more than 1 year. Globally, most schools and public places have been temporarily closed. In several countries, students have been instructed to stay at home, engage in social distancing during daily activities, and study from home (1). They used various Internet devices to stay connected to their schools and peers. Consequently, their screen time may have increased, especially of those living in urban cities in Indonesia. Internet access among adolescents has been estimated at 79.5%, and most of them use the Internet for several purposes such as to seek information for school activities, connect with their peer groups through social media networking (such as Path, Line, Whatsapp, Facebook messenger, etc.), and for entertainment (2). Adolescents have missed several important milestones, including direct social interactions with peers, making new friends, and sports activities, among others (1, 2). Moreover, they have been forced to adapt to new educational styles (i.e., online learning, examination[s], and group work), especially students in their final year of high school. Students entering the new academic year, particularly if they have transitioned to a new school, can only meet their new friends or teachers via online classes (3, 4). Therefore, their screen time has been increased compared to before the COVID-19 pandemic. It may increase the risk of cyberbullying for victims (5, 6). Moreover, adolescents may lose peers and social support, face more internal conflicts, and abuse triggered by less personal space because all family members stay at home, engage in less physical activity due to parental restrictions to leave the home, and disruptions in daily routine activities (7). Additionally, the number of COVID-19 cases and deaths has continued to increase in several countries, including India, Indonesia, the United States, and Brazil (8). In Indonesia, the number of new COVID-19 cases has increased over time. For example, in this study period (August-October 2020), the average number of new cases was estimated to be around 4,200 per day and around 100 deaths occurred due to this infection. However, it increased by 10–15 times in the middle of July 2021, with more than one thousand deaths. The Indonesian government has implemented several policies to minimize the spread of COVID-19 since April 2020, such as extending the policy of restricting public activities at the micro scale and school closures, implementing a 50–100% Work-From-Home policy, while all shopping centers and malls, worship houses, and public parks were partially or totally closed (2, 9). Thus, the COVID-19 pandemic has significantly contributed to stressful life events among adolescents and young adults. It has become a unique stressor and forces everyone, including adolescents, to rapidly acquire new adjustment skills. However, adolescents may face difficulties due to developmental challenges.

Adolescence is a transitional period between childhood and young adulthood, consisting of multidimensional transformations such as biological, psychological, cognitive, and social. From a biopsychosocial perspective, maturation of the hypothalamic-pituitary-gonadal axis marks both puberty and adolescence. Thus, hormonal changes in adolescence are associated with fluctuations in emotional experiences, increased self-esteem, a sense of self-importance, and individuality. However, at the same time, adolescents may experience self-criticism, depression, anxiety, and anger (10). Additionally, prefrontal cortex immaturity may contribute to irrational decision-making and tenuous impulse control, particularly during stressful times (11). Moreover, this can lead to greater experimentation with high-risk behaviors during the COVID-19 pandemic, such as non-suicidal self-injury (NSSI), not only among typical adolescents but also among adolescents with pre-existing mental health problems (12, 13).

In 2018, the International Society for the Study of Self-Injury defined NSSI as conscious and direct engagement in behaviors that produce body tissue damage without deliberate suicidal intention (1). However, NSSI is linked to suicidal ideation and attempt(s), and it has been estimated that individuals with NSSI are four times more likely to attempt suicide in the future (14, 15). In 2015, Plener et al. reported that NSSI typically began during early or mid-adolescence, possibly becoming chronic NSSI over a prolonged period or was carried out a few times in a significantly more discreet period (16, 17). A worldwide study demonstrated that ~17% of adolescents engaged in NSSI, and it was essentially comparable in boys and girls (18, 19). Moreover, cultural differences may impact the prevalence of NSSI, despite the paucity of prevalence studies in non-Western countries compared to Western countries (20). In the past decade, many mental health-related studies have investigated NSSI. Previous studies have indicated that gender, cyberbullying, screen time, abuse, and stress may be associated with NSSI, especially during the unconventional life events of the COVID-19 pandemic (6–8). On the other hand, several studies before the COVID-19 pandemic revealed other psychosocial and biological risk factors associated with NSSI, such as genetics, changes in brain neurotransmitters, depressive symptoms, stigmas, common misperceptions of mental illness, and family discord (20).

NSSI can be characterized as an improper coping strategy for adolescents, especially to release strong negative feelings due to heightened stress and relief from their intolerable states in a very short time (13). Furthermore, NSSI may predict poorer future psychosocial well-being among adolescents (1). Several studies have reported that closure of schools and public places during the COVID-19 pandemic magnified many negative consequences of adolescents' developmental milestones and possibly increased the risk for NSSI or exacerbated self-injury behavior such as suicidal ideation or attempt in some adolescents due to elevated stress during this unstable period (1, 21). To summarize, NSSI has possibly become a new threat to adolescents' mental well-being during the COVID-19 pandemic. Therefore, the effect of screen time, cyberbullying, abuse, and stress on NSSI that emerged during the COVID-19 pandemic is a critical topic to be investigated and discussed.

The current study constructed and empirically tested a theoretical model that could predict and explain adolescent NSSI during the COVID-19 pandemic in Indonesia. Several studies have reported that NSSI has become a major public health problem worldwide during the COVID-19 pandemic, especially among adolescents (13, 20). However, to the best of our knowledge, few empirical studies have explored the effects of cyberbullying, abuse, screen time, and stress on adolescent NSSI during the COVID-19 pandemic. Accordingly, the current study developed a questionnaire for adolescents in Indonesia. The questionnaire survey method was adopted to determine how cyberbullying, screen time, abuse, and stress affected adolescent NSSI during the COVID-19 pandemic. Hence, the present study explored several primary antecedents/predictors (cyberbullying, screen time, and abuse) for NSSI among adolescents, and determined whether stress mediated cyberbullying, screen time, and abuse of adolescent NSSI. In this context, the research questions were defined as follows: Is there any possibility that mental health reactions during the COVID-19 pandemic, such as cyberbullying, screen time, and abuse, significantly predict adolescent NSSI? How does stress mediate the effects of cyberbullying, abuse, and screen time on adolescent NSSI during the COVID-19 pandemic? The results were expected to enhance the scholarly understanding of adolescent NSSI during the COVID-19 pandemic and adolescents' mental health reactions, such as cyberbullying, screen time, abuse, and stress, to enhance the knowledge base for health professionals, parents, schools, and government education authorities, and design better adolescent mental health and stress reduction programs, especially during the pandemic.

## Methods

This cross-sectional study used a “crowdsourcing” sample collection method during the COVID-19 pandemic in Indonesia. Data were collected from August 21 to October 10, 2020. A questionnaire link (http://surveymonkey.com) was circulated through social media networks, such as WhatsApp, Facebook Messenger, and Line. The inclusion criteria were adolescents aged 11–17 years with secondary and high school backgrounds. Before completing the survey, participants completed an online informed consent form. During the research period, 744 questionnaires were returned. However, 247 were excluded because they were incomplete, including missing data, not fulfilling the inclusion criteria, or the absence of a signature on the online informed consent form. Therefore, the final analysis included 464 questionnaires. The Ethics Committee of the Faculty of Medicine of Universitas Indonesia approved the study protocol in April 2020 (KET-375/UN2.F1/ETIK/ PPM.00.02/2020).

### Instruments

The questionnaire was developed specifically for this study. It contained information from a previously published study with multi-item scales that demonstrated good psychometric properties. The questionnaire items were determined after a thorough review of several relevant studies that addressed cyberbullying, screen time, abuse, stress, and NSSI. The item dimensions were modified to fit the context of adolescent mental health reactions during the COVID-19 pandemic and study design. Questionnaire development followed the recommendations of MacKenzie et al. and the development procedures suggested by Devellis for standard psychometric scales (22, 23). The questionnaire consisted of 24 items measured in six sections: the cyberbullying scale (three items), screen time scale (three items), abuse scale (three items), stress scale (seven items), NSSI scale (three items), and sociodemographic questions. All questions were modified into the Indonesian language and had good construct reliability (CR ranged from 0.759 to 0.955) for this study.

#### Cyberbullying Scale

The cyberbullying scale was developed using three items modified from Patchin and Hinduja (24), Sourander et al. (25), and Hinduja et al. (26), Sourander et al. (25), and Wiguna et al. (27). The questions were as follows: “During the past 6 months, how often have you been cyber-bullied?” “During the past 6 months, how often have you cyber-bullied others?” “During the past 6 months, how often have you been cyber-bullied and being cyber-bullied others?” Items were rated on a four-point Likert scale, scored as follows: never = 1, < 1 per week = 2, > 1 per week = 3, and almost daily = 4. The construct reliability (CR) of these three items was 0.958, which was satisfactory in terms of measuring the constructs of interest because it exceeded 0.5.

#### Screen Time Scale

Screen time was measured using three items modified from the Youth Screen Time Survey. The method used to measure screen time in this study followed the standard methods used in several other peer-reviewed studies. Adolescents were asked to report the number of minutes devoted to the following three typical activities on weekdays and weekends: (1) “television (movies/videos/YouTube, playing console/video games),” (2) “using personal computers (such as, laptops/tablets/iPads either for browsing, YouTube, and/or social media activities),” and (3) “smartphone devices (for online games, browsing, social media connections, and/or online shopping).” The daily time spent on each screen time activity was calculated by averaging the weekday and weekend screen times of the three typical activities and dividing by 7. The average total weekday and weekend screen time for each type of activity was divided by 7 and categorized into a six-point Likert scale as follows: < 2 h = 1, 2–4 h = 2, 4–6 h = 3, 6–8 h = 4, 8–10 h = 5, and ≥ 10 h = 6. The CR for screen time measurement was 0.759, which was satisfactory in terms of measuring the constructs of interest.

#### Abuse Scale

The construct of abuse was measured using three items from the Adverse Childhood Experiences (ACE) scale in the CDC-Kaiser Permanente Adverse Childhood Experiences Study (28). The three modified questions were as follows: “In the past 3 months, did a parent or other adult in the household often or very often push, grab, slap, or throw something at you? or ever hit you so hard that you had marks or were injured?” “In the past 3 months, did an adult or person at least 5 years older than you ever swear at you, insult you, or put you down?” and “In the past 3 months, did you often or very often feel that you did not have enough to eat, had to wear dirty clothes, and had no one to protect you? or your parents or anybody else in your home were not taking good care of you or giving you enough love as you needed?” The items were scored as yes = 1 and no = 2. The CR of the abuse scale was 0.955, which was satisfactory in terms of measuring the constructs of interest.

#### Stress Scale

The stress scale was developed using seven items modified from the Depression Anxiety Stress Scale-21 (DASS-21) (29). It is a quantitative measure of the general symptoms of stress in the past 7 days across clinical, community, and non-clinical samples (30–32), and different countries, cultures, and languages (33–36). The seven modified items consisted of the following: “I find myself getting upset because of minor issues,” “I have a tendency to over-react to different situations,” “I find it is hard to relax,” “I find myself easily getting upset,” “I feel that I am using a lot of energy to feel worry,” “I find myself getting impatient when something needs to be postponed (i.e., queuing, waiting for class, traffic jams, etc.),” and “I am easily getting irritated.” Items were scored on a four-point Likert scale, as follows: never = 1, sometimes = 2, always = 3, and almost always = 4. The CR of the stress scale was 0.766, which was satisfactory in terms of measuring the constructs of interest.

#### NSSI Scale

Non-suicidal self-injury (NSSI) was measured using three items modified from Wiguna et al. and Sourander et al. (25, 27). The modified questions were as follows: “In the past 6 months, did I ever hurt myself deliberately, such as intentionally self-injured,” “In the past 6 months, did I ever seriously consider killing myself,” and “In the past 6 months, did I ever try to defeat myself.” The items were scored as yes = 1 and no = 2. The CR of the NSSI scale was 0.953, which was satisfactory for measuring the constructs of interest.

#### Sociodemographic Questions

The sociodemographic questions included eight items that inquired about the participants' age, sex, level of education, home-based and parental socioeconomic background. The questions were designed for nominal and categorical responses.

#### Data Analysis

**Figure 1:**
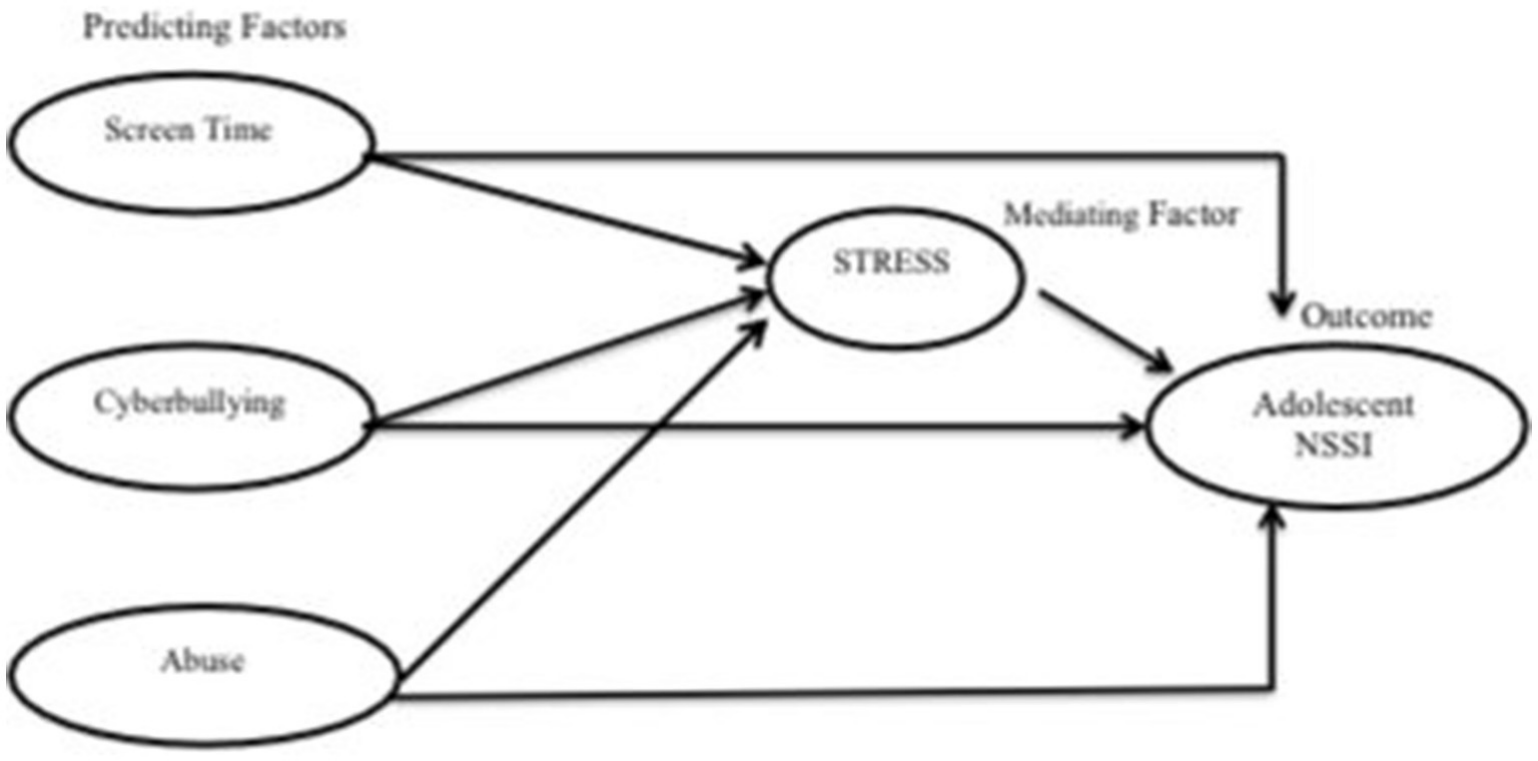
The pattern of construct interaction pathway of predictors (screen time, cyberbullying, abuse), stress as the mediating variable, and adolescents' NSSI as the dependent variable (outcome).

The present study determined the primary predictors of NSSI (screen time, abuse, and cyberbullying) and confirmed that stress had a mediating effect on these predictors of adolescent NSSI during the COVID-19 pandemic. Therefore, structural equation modeling (SEM) analysis was performed using Lisrel version 8.8. The SEM analysis primarily aimed to explain the pattern of construct interaction pathways of several inter-related independence predictors simultaneously (screen time, cyberbullying, and abuse), stress as the mediating variable, and adolescent NSSI as the dependent variable or outcome (Figure 1). The SEM analysis is a strong technique for effectively addressing multicollinearity (when ≥ 2 variables are highly associated), which is one of the advantages of SEM over multiple regression and factor analysis. The mediating effect of stress can be described as follows: full mediation (a mediator fully explains the interaction of the predictor variable to predict the outcome, and there is no relationship without the mediator in the model), partial mediation (predictor variable has a direct significant interaction to predict the outcome, even when the mediator is removed from the model; the mediator only partially explained the inter-relationship), or no mediation (predictor variable does not have any direct significant interaction to predict the outcome and is not statistically significant even when the mediator is included). The construct interaction pathway to predict the adolescent NSSI in this study was considered to be statistically significant if each predictor and mediator exceeded the critical value of indicator's loading for *p* < 0.05 (critical *t*-value for the path >1.96) (37, 38).

Lisrel version 8.8 was used to analyze CR, which reflects the internal consistency reliability of the variable measurement scale. Reisinger and Turner reported that the CR coefficient of each measurement scale should be ≥ 0.6 as a prerequisite for further SEM analysis (36). The obtained CR coefficient in this study ranged from 0.759 for the screen time scale to 0.958 for the cyberbullying scale, and all CR coefficients were > 0.6 (Table 1). Hence, the reliability of all scales in the present study was acceptable for further SEM analysis using Lisrel version 8.8.

**Table 1:** The construct reliability of the scales.

**Construct**	**Items**	**Mean (SD)**	**Construct reliability (CR)**
Cyberbullying	3	3.44 (1.56)	0.958
Abuse	3	4.34 (0.66)	0.955
Screen time	3	8.34 (3.21)	0.759
Stress	7	14.41 (4.64)	0.766
NSSI	3	3.5 (0.78)	0.953

## Results

This study included 464 adolescents selected using a crowdsourcing sample collection method. The mean (± SD) age of the cohort was 14.61 ± 1.65 years, and consisted of 66.7% females. Secondary high school was the highest educational background (58%), followed by senior high school (38.8%) and elementary school (3.2%). Participants were from several provinces in Indonesia; the greatest proportion was from Jakarta (68.5%), followed by West Java (10.6%), Banten Province (6.7%), East Java (4.5%), Central Java (2.8%), and Sumatra (2.5%). Most participants had parents with middle to high economic backgrounds (83.8%), while the remainder had a lower economic background (16.2%) (Table 2).

**Table 2:** Characteristic of research subjects (*n* = 464).

**Characteritics**	***n* (%)**	**Mean (SD)**	**Median (Range)**	**95% Confidence Interval of the mean**
**Age**	464 (100)	14.61 (1.65)	14 (11–17)	14.45–14.76
**Gender**				
Boys	155 (33.4)			
Girls	309 (66.6)			
**Home-based**				
Jakarta	318 (68.5)			
West Java	49 (10.6)			
Central Java	13 (2.8)			
East Java	21 (4.5)			
Banten	31 (6.7)			
Yogyakarta	6 (1.3)			
Bali	2 (0.4)			
Sumatera	12 (2.5)			
Kalimantan	12 (2.5)			
**Educational background**				
Elementary school	15 (3.2)			
Secondary high school	269 (58)			
Senior high school	180 (38.8)			
**Parental socio-economic level**				
Low level income	75 (16.2)			
Moderate level income	183 (39.4)			
High level income	206 (44.4)			

**Figure 2:**
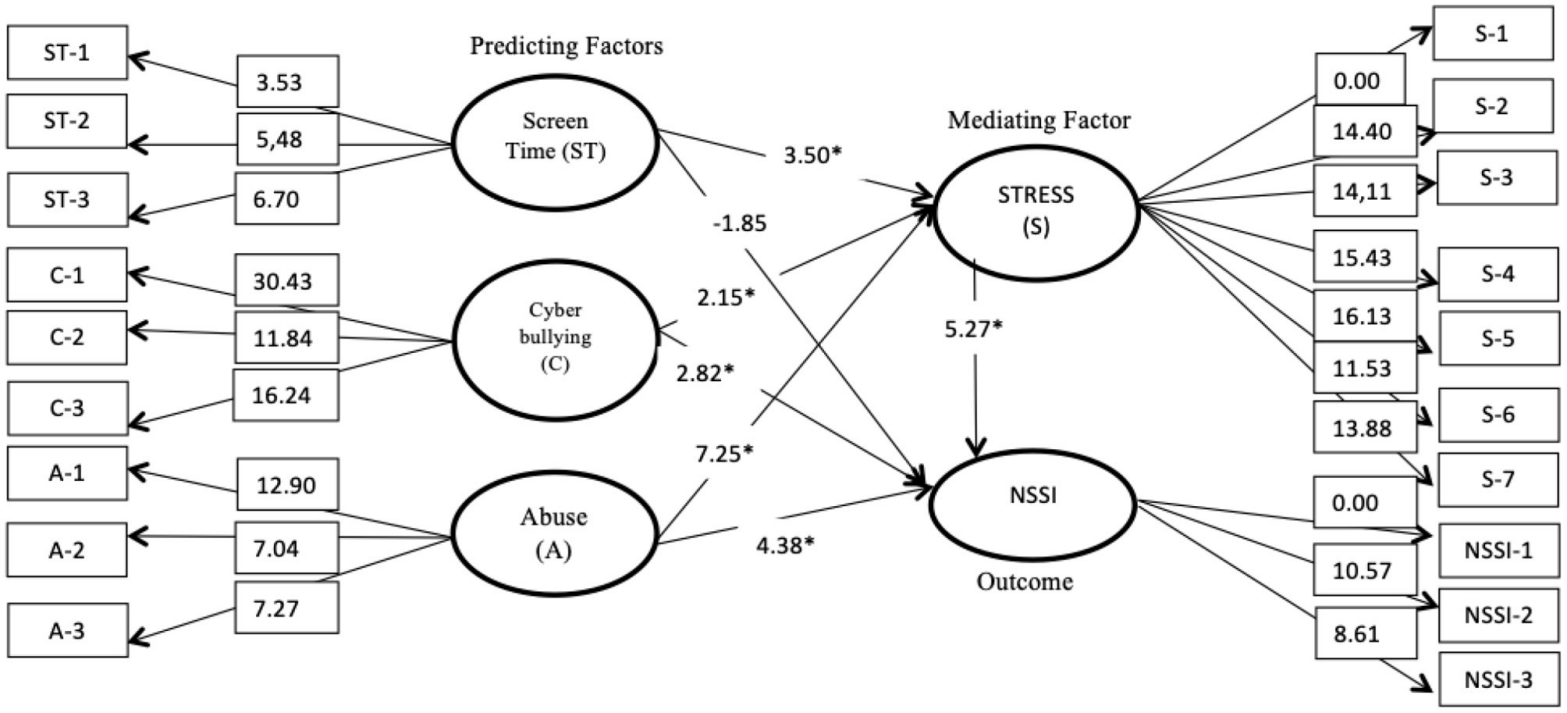
The construct theoretical model of adolescent NSSI based on the SEM analysis including the critical *t*-value (Statistically significant on critical *t*-value >1.96 and *p* < 0.05). The analysis showed that stress significantly and fully mediated the relationship between screen time and adolescent NSSI. However, it only had a partial significant mediating effect on cyberbullying and abuse. The theoretical construct analysis was significantly fit to the model. *p < 0.05; Root Mean Square Error of Approximation = 0.038, Normed Fit Index = 0.95, Comparative Fit Index = 0.98, Relative Fit Index = 0.94, Non-Normed Fit Index = 0.98, Standardized Root Mean Square Residual = 0.049, Goodness-of-Fit Index = 0.95, Adjusted Goodness-of-Fit Index = 0.93).

Results demonstrated that cyberbullying and abuse became significant positive direct predictors of adolescent NSSI during the COVID-19 pandemic (critical *t*-value for cyberbullying: 2.82; critical *t*-value for abuse: 4.38). However, screen time was not a direct predictor of adolescent NSSI (critical *t*-value: 1.85). Additionally, stress had a significant mediating effect on cyberbullying, screen time, and abuse in the relationship with adolescent NSSI (critical *t*-value: 5.27). Stress also had a full mediating effect on screen time in predicting adolescent NSSI, but only had a partial mediating effect on cyberbullying and abuse. Furthermore, cyberbullying, screen time, and abuse without the mediating effect of stress predicted 38% (*R*^2^ = 0.38) of the variance in adolescent NSSI. Meanwhile, cyberbullying, screen time, and abuse with the mediating effect of stress could explain 48% of the variance in adolescent NSSI (*R*^2^ = 0.48) (Table 3). Thus, stress as a mediator variable significantly multiplied the interaction between predictors (cyberbullying, screen time, and abuse) to predict adolescent NSSI during the COVID-19 pandemic in Indonesia (Figure 2). The mediating effects of stress were determined according to the significance of the interaction model (critical *t*-value for the path >1.96; *p* < 0.05). Hence, the research questions (i.e., Is there any possibility that mental health reactions during the COVID-19 pandemic, such as cyberbullying, screen time, and abuse, significantly predict adolescent NSSI? How does stress mediate the effects of cyberbullying, abuse, and screen time on adolescent NSSI during the COVID-19 pandemic?) were answered based on the SEM analysis.

**Table 3:** Structural equation modeling (SEM) analysis results.

**Construct**	**Model 1**		**Model 2**	
	**β**	**Critical *t*-value**	**β**	**Critical *t*-value**
Cyberbullying→ NSSI	0.14	2.82^*^	0.10	2.15^*^
Abuse→ NSSI	0.35	4.38^*^	0.47	7.25^*^
Screen time→ NSSI	−0.12	−1.85	0.22	3.50^*^
*Mediating effect*				
Stress→ NSSI			0.41	5.27^*^
*R*^2^ NSSI	0.35		0.48	

* *p-value 0.05*.

The structural equation path modeling in this study followed the recommendations of Turner and Reisinger (37) and Muller et al. (38) to select goodness-of-fit to measure the fitness of the structural model (37, 38). Based on confirmatory factor analysis of the structural path modeling, the construct showed a significant model of fitness (root mean square error of approximation = 0.038; normed fit index = 0.95; comparative fit index = 0.98; relative fit index = 0.94; non-normed fit index = 0.98; standardized root mean square residual = 0.049; goodness-of-fit index = 0.95; and adjusted goodness-of-fit index = 0.93).

## Discussion

The COVID-19 pandemic is ongoing, and mental health consequences cannot be avoided by everyone. Adolescence is a period of turmoil, as young people seek independence and struggle to find their identity. The major developmental task during this period is to improve social skills, become empathetic individuals, and find their true identity. These developmental tasks arise through connections with peers. Therefore, disrupted connections with social contexts and peers may have several implications for their mental well-being. Moreover, the immaturity of the prefrontal cortex during adolescence may make these individuals significantly more vulnerable to various mental health consequences during this pandemic (11).

During the COVID-19 pandemic, the lack of direct social connectedness with peers due to stay-at-home policies may have strengthened feelings of loneliness and social isolation. Therefore, adolescents alternatively engaged in more screen time during their daily activities (1). This study revealed that screen time did not directly predict adolescent NSSI, but demonstrated that stress was a perfect mediating factor of screen time and adolescent NSSI. Screen time is defined as the quantity of time spent and the diverse activities performed online using digital devices (39). This study found that the average screen time was around 4–6 h per day and was used for (1) watching television (such as movies/videos/YouTube and/or playing console/video games), (2) personal computers (such as laptops/tablets/iPads for browsing, YouTube, and/or social media activities), and (3) smartphone devices (for online games, browsing, social media connections, and/or online shopping). We found that the average screen time among adolescents was in keeping with the screen time prescribed by the American Academy of Child and Adolescent Psychiatry (from the recommended hours to more than 6 h). Hamilton et al. explained that an appropriate amount of screen time devoted to social media or other activities may act as a protective factor for mental health among adolescents because it can provide appropriate physical and mental health information, academic materials, maintain social connections with peers, and facilitate self-expression (40). On the other hand, several studies have focused on the potentially harmful mental health effects of increased screen time, such as high exposure to false or misinformation on physical and mental health related to the COVID-19 pandemic, cyberbullying, and age-inappropriate media programs. Moreover, many adolescents may have insufficient basic knowledge, understanding, and perception to assess the accuracy of this information because of prefrontal cortex immaturity (38–41). Thus, the cognitive processes that follow screen time for television, personal computers, or smartphones may trigger adolescents to feel stressed, leading them engage in NSSI behavior to cope with these uncomfortable feelings.

Interestingly, stress was found to be a partial mediating factor for cyberbullying and abuse (as a predictor factor) of NSSI. Cyberbullying and abuse directly and significantly predicted adolescent NSSI; however, the association was more significant through the mediator effect (stress). Cyberbullying is defined as a type of bullying exerted through devices such as computers, laptops, or smart phones on Internet and social media applications (25). In 2012, Langos explained that it can occur either directly or indirectly depending on privileged or public posts, including negative content that embarrasses others on private text messages or pictures through social media platforms (Whatsapp, Path, and Line, or private e-mail) (42). Extensive Internet use during the COVID-19 pandemic can heighten loneliness and impulsive behaviors toward other adolescents, such as domestic abuse, cyberbullying, and other high-risk and self-injurious behaviors (43–45). In 2018, Wiguna et al. found that cyberbullying increased the risk for high-risk behaviors such as self-harm, suicidal ideation, and attempted suicide (25). This process is related to brain networks. First, the socio-emotional network reacts to the reward processing part of the brain and subtle emotional stimuli. The second network is cognitive control, which plays an important role in planning, rational thinking, and self-regulation. During adolescence, the socio-emotional network becomes more dominant compared to the cognitive control network due to the immaturity of the prefrontal cortex (46). Hence, adolescents may be more easily engaged in impulsive and hostile behaviors.

The COVID-19 pandemic is a global crisis affecting every sector of life, such as health, economies, and family quality of life (47). Thus, it may cause disruptions in many family systems, not only due to lockdowns, stay-at-home orders, school from home, social distancing, and difficulties with access to health services, but also because of the sudden and possibly long-term family poverty and uncertainty (48, 49). Moreover, Martinkevich et al. explained that pandemics produce a deviant situation in which adolescents' socio-ecological systems are disrupted and, consequently, the incidence of abuse is likely to increase (50). The socio-ecological model explained that the COVID-19 pandemic may alter adolescents' cognition, emotions, behaviors, and fundamental mechanisms due to limited access to their developmental needs. Hence, these mutual interaction processes manifest as changing psychological, interpersonal, well-being, and environments, and in the ways in which adolescents adapt to and modify these environments (51). At the microsystem level, it may possibly increase oppositional and impulsive behaviors and limit testing among adolescents. This hostile behavior may elicit punitive responses from parents (52). They may also experience parental burnout, either constrained or worsened by the consequences of the pandemic. Adolescents' own stress and uncertainty regarding the pandemic may worsen the feeling of tension and they may become violent toward themselves due to limitations in their capacity to make decisions (53).

The study findings revealed that stress significantly mediated the predictors and, thus, the effects on NSSI behavior were more heightened. Liu and Miller (54) reported that stress is theoretically and empirically associated with an increased risk of self-injury, particularly in the form of suicidal ideation and behavior (55). The finding that stress possibly acts as a primary mediator of adolescent NSSI was supported by several theoretical conceptualizations that originally came from the two distinct processes with four functional elements of NSSI (56). NSSI behavior was strengthened by two distinct processes that consisted of four functional elements: positive and negative reinforcements in intrapersonal emotional regulation processes and interpersonal function processes. Intrapersonal emotional regulation processes include negative reinforcement that releases the conflict or a decrease in the negative affect following engagement in NSSI and positive reinforcement that is involved in the urge to feel pain or act on the feelings of guilt through self-punitive behavior. Interpersonal function processes include positive reinforcement wherein NSSI serves as a means of communicating with the unconscious mind for help and support and negative reinforcement that interrupts negative interpersonal interactions following NSSI. Such interpersonal functions may be relevant to adolescents because of the immaturity of brain's cognitive networks that impair interpersonal problem-solving skills and deprive them of general communication abilities (54, 57, 58). Nevertheless, stress during the COVID-19 pandemic became a mediating presence of a form of distress across the four functional elements, which could be the reason behind adolescents' engagement in NSSI to cope with this distress.

Based on the study findings, expanding adolescent mental health programs that can promote better coping strategies to manage stress related to cyberbullying, abuse, and increased screen time, such as coping with stress and positive attitudes toward stress, may be redesigned to ensure adequate self-adjustment during this pandemic. Furthermore, adolescent stress-reduction programs may be developed to improve coping strategies on these difficult days. Adolescent mental health programs are usually conducted at schools because adolescents spend most of their time at school. However, during the pandemic period, this program may be conducted online. Hence, adolescent mental health intervention programs that promote effective coping strategies to manage stress during the COVID-19 pandemic, such as active solution-orientation, stress resolution, conflict with stress, mindfulness, and positive attitude instead of holding back problems to one self may be designed to ensure adequate emotional adjustment (59). Several studies reported that sufficient regulation of emotions, including correct problem-solving skills and creating positive emotions in daily life through shared actions using networks and information and communication technologies (ICT) were very helpful to reduce stress during this COVID-19 pandemic (60, 61). Alternatively, psychoeducation programs may be developed in a very small group of adolescents in a “safe-haven” environment to enhance coping strategies and emotional regulation toward stress (62). Even for adolescents learning from home, schools should strive to intensify social support, encouragement, reassurance, and offer mental health services and programs, especially to those with existing mental health issues that enhance their vulnerability to stress (63).

Nevertheless, this study had several limitations. First, other factors that may be associated with adolescent NSSI, such as genetics, parenting, the role of devices, subjective feelings during the COVID-19 pandemic, and previous mental health history were not addressed. Therefore, further studies can elaborate these using a similar model. Second, the questionnaire relied on adolescents' self-reporting recruited through crowdsourcing, which may introduce biases related to misunderstanding or misreporting to avoid stigma and forgetting experiences that have already happened that triggered the recall/response bias. However, the study minimized these biases by providing detailed text explanations before the participants started the survey and provided a detailed explanation of each question. Third, the study was conducted online and may have only covered adolescents with access to the Internet. Future studies may be designed with mixed method data collection (i.e., online and offline surveys) so that it can include more adolescents, especially those without access to the Internet. Data were collected from August to early October 2020. This period was considered to be the second wave of the COVID-19 pandemic, and it came together with the beginning of the new online academic semester; therefore, the participants may have been overwhelmed. Future studies should consider the time period required for data collection to reduce unpredictable bias. Meanwhile, the study was conducted with a cross-sectional design, and it may not reflect the cause-and-effect relationship. Therefore, future studies can be designrd to determine the cause-effect relationship between adolescent NSSI, stress, cyberbullying, and other related factors.

To our knowledge, this study was the first in Indonesia and, perhaps, in Southeast Asia, to construct and analyze a theoretical model that can predict and explain adolescent NSSI during the COVID-19 pandemic. Stress was found to be a mediating factor in the relationship of adolescent NSSI with screen time, cyberbullying, and abuse. Therefore, the theoretical model can be applied further to design adolescent mental health programs, especially those associated with coping with stress in daily life.

## Data Availability Statement

The original contributions presented in the study are included in the article/Supplementary Material, further inquiries can be directed to the corresponding author/s.

## Ethics Statement

The studies involving human participants were reviewed and approved by the Ethics Committee of the Faculty of Medicine of Universitas Indonesia. Informed consent to participate in this study was provided by the study participants.

## Author Contributions

TW: designing, analyzing, and writing the results and discussion. KM: designing, writing discussion, and results analyzing. FK and RI: designing and writing discussion. EW: results analyzing and wrting discussion. BM: designing and results analyzing. KP: editing the document and contributing in the table analyzing. All authors contributed equally for this paper.

## Funding

The publication was supported by the PUTI Grant Universitas Indonesia with contract number NKB-4136/UN2.RST/HKP.05.00/2020. The funder did not have any involvement in the study design or report writing.

## Conflict of Interest

The authors declare that the research was conducted in the absence of any commercial or financial relationships that could be construed as a potential conflict of interest.

## Publisher's Note

All claims expressed in this article are solely those of the authors and do not necessarily represent those of their affiliated organizations, or those of the publisher, the editors and the reviewers. Any product that may be evaluated in this article, or claim that may be made by its manufacturer, is not guaranteed or endorsed by the publisher.
