# Supplementary material for: The Effect of Cyberbullying, Abuse, and Screen Time on Non-suicidal Self-Injury Among Adolescents During the Pandemic: A Perspective From the Mediating Role of Stress
Source: Front Psychiatry. 2021 Nov 12;12:743329. doi: 10.3389/fpsyt.2021.743329 (PMC8632872; doi:10.3389/fpsyt.2021.743329)
Supplement: Supplementary file 1 [file Data_Sheet_1.PDF]

Questionnaire

1. Demographic Characteristic
    a. Age : …….years
    b. Gender : Male/Female
    c. Educational background: Senior High-school/Junior High-school, grade…..
    d. Home-based in Indonesia: ……
    e. Parental socio-economic background: Rp. ……… per month

2. Cyberbullying Scale

No.	Item	Never	< 1 per week	> 1 per week	Almost daily
1.	During the past six months, how often have you been cyber-bullied?				
2.	During the past six months, how often have you been cyber-bullied and being cyber-bullied others?				
3.	During the past six months, how often have you cyber-bullied others?				

3. Screen Time Scale
   In weekdays how many minutes do you:
    a. Watching television (movies/videos/YouTube, playing console/video games) ….. minutes
    b. Using personal computers (such as, laptops/tablets/iPads either for browsing, YouTube, social media activities) ….. minutes
    c. Using smartphone devices (for online games, browsing, social media connections, online shopping) …. Minutes

   In week-end how many minutes do you:
    a. Watching television (movies/videos/YouTube, playing console/video games) ….. minutes
    b. Using personal computers (such as, laptops/tablets/iPads either for browsing, YouTube, social media activities) ….. minutes
    c. Using smartphone devices (for online games, browsing, social media connections, online shopping) …. Minutes

4. Abuse Scale

No.	Item	Yes	No
1.	In the past three months, did a parent or other adult in the household often or very often push, grab, slap, or throw something at you?<br><br>Or ever hit you so hard that you had marks or were injured?		
2.	In the past three months, did an adult		

	or person at least 5 years older than you ever swear at you, insult you, or put you down?		
3.	In the past three months, did you often or very often feel that you did not have enough to eat, had to wear dirty clothes, and had no one to protect you?<br><br>Or your parents or anybody else in your home were not taking good care of you or giving you enough love as you needed?		

5. Stress Scale

No.	Item	Never	Sometimes	Always	Almost always
1.	I find myself getting upset because of minor issues				
2.	I have a tendency to over-react to different situations				
3.	I find it is hard to relax				
4.	I find myself easily getting upset				
5.	I feel that I am using a lot of energy to feel worry				
6.	I find myself getting inpatient when something needs to be postponed (i.e., queuing, waiting for class, traffic jams, etc0				
7.	I am easily getting irritated				

6. NSSI Scale

No.	Item	Yes	No
1.	In the past six months, did I ever hurt myself deliberately, such as intentionally self-injured		
2.	In the past six months, did I ever seriously consider killing myself		
3.	In the past six months, did I ever try to defeat myself		
